# Within-Otolith Variability in Chemical Fingerprints: Implications for Sampling Designs and Possible Environmental Interpretation

**DOI:** 10.1371/journal.pone.0101701

**Published:** 2014-07-07

**Authors:** Antonio Di Franco, Fabio Bulleri, Antonio Pennetta, Giuseppe De Benedetto, K. Robert Clarke, Paolo Guidetti

**Affiliations:** 1 Université de Nice Sophia-Antipolis, Faculté des Sciences, EA 4228 ECOMERS, Nice, France; 2 Laboratory of Conservation and Management of Marine and Coastal Resources, Dipartimento di Scienze e Tecnologie Biologiche ed Ambientali (DiSTeBA), University of Salento-CoNISMa (Consorzio Nazionale Interuniversitario per le Scienze del Mare), Lecce, Italy; 3 Dipartimento di Biologia, Università di Pisa, Pisa, Italy; 4 Laboratorio di Spettrometria di massa analitica ed isotopica, Dipartimento di Beni Culturali, University of Salento, Lecce, Italy; 5 Plymouth Marine Laboratory, Prospect Place, West Hoe, Plymouth, United Kingdom; University of Auckland, New Zealand

## Abstract

Largely used as a natural biological tag in studies of dispersal/connectivity of fish, otolith elemental fingerprinting is usually analyzed by laser ablation-inductively coupled plasma-mass spectrometry (LA-ICP-MS). LA-ICP-MS produces an elemental fingerprint at a discrete time-point in the life of a fish and can generate data on within-otolith variability of that fingerprint. The presence of within-otolith variability has been previously acknowledged but not incorporated into experimental designs on the presumed, but untested, grounds of both its negligibility compared to among-otolith variability and of spatial autocorrelation among multiple ablations within an otolith. Here, using a hierarchical sampling design of spatial variation at multiple scales in otolith chemical fingerprints for two Mediterranean coastal fishes, we explore: 1) whether multiple ablations within an otolith can be used as independent replicates for significance tests among otoliths, and 2) the implications of incorporating within-otolith variability when assessing spatial variability in otolith chemistry at a hierarchy of spatial scales (different fish, from different sites, at different locations on the Apulian Adriatic coast). We find that multiple ablations along the same daily rings do not necessarily exhibit spatial dependency within the otolith and can be used to estimate residual variability in a hierarchical sampling design. Inclusion of within-otolith measurements reveals that individuals at the same site can show significant variability in elemental uptake. Within-otolith variability examined across the spatial hierarchy identifies differences between the two fish species investigated, and this finding leads to discussion of the potential for within-otolith variability to be used as a marker for fish exposure to stressful conditions. We also demonstrate that a ‘cost’-optimal allocation of sampling effort should typically include some level of within-otolith replication in the experimental design. Our findings provide novel evidence to aid the design of future sampling programs and improve our general understanding of the mechanisms regulating elemental fingerprints.

## Introduction

Otoliths (also called earstones) are paired calcified structures with a function in balancing and hearing in all teleost fishes [1]. Otoliths are composed of aragonite deposited on a proteinaceous matrix and grow by deposition of daily and annual increments throughout the life of the fish [2]. This property allows otoliths to be used in estimating fish age (see [3] and references therein).

Otoliths, while growing, incorporate into their calcium carbonate matrix both minor and trace elements. Some elements (e.g. Sr and Ba) are incorporated at rates related to their environmental concentrations [4], so that their concentration reflects local availability in the seawater [2]. Uptake of other elements (e.g. K, Na, Zn, Mn) is likely to be mediated by physiological regulation [1], [5]. Regardless of the mechanism regulating incorporation into the otoliths, an elemental fingerprint (i.e. the otolith elemental composition) can be used as a permanent spatial signature of the marine environment experienced by a fish during the various phases of its life cycle [1], [2], [6], [7]. Otoliths are, therefore, a natural biological tag for investigating dispersal and connectivity patterns of fish and delineating stocks/populations [1], [5], [8], [9]. Understanding the spatial structure of fish stocks and the connectivity between them is increasingly considered a crucial requirement for sustainable fisheries management [10].

Otolith chemistry has been widely used to address a variety of ecological and conservation issues (e.g. designing networks of marine protected areas, see [11]). This explains the rapid growth of this methodology reported in the scientific literature, with 6 papers published prior to 1980, 157 by the end of 1998 [2], 700 by 2011 [10] and more than 1300 published by 2013 (from ISI Web of Knowledge; Topic =  ((chemist* or chemical or elemental) and otolith), Timespan = 1950–2013).

A basic requirement to use otolith chemistry as a biological tag is a sounded assessment of spatial variation in the elemental fingerprint [2], [12]. In otolith fingerprint studies, as in any ecological study, assessing spatial variability at multiple scales is considered a first step in: i) inferring the mechanisms and processes possibly driving the observed patterns; ii) identifying the most appropriate/relevant scales of sampling [13], [14]. The identification of relevant scales of variation is thus a prerequisite for proposing and testing explanatory models, as different processes are likely to operate at different spatial (and temporal) scales [15]. For this purpose, a number of studies have measured spatial and temporal variation in a multitude of different biological and environmental contexts using hierarchical analyses of variance [16], [17].

In order to analyze elemental fingerprints, a large suite of techniques and technologies has been adopted (see [1]) but, so far, the most common method utilizes laser ablation-inductively coupled plasma mass spectrometry (LA-ICP-MS, [18]). This technique allows the analysis of different otolith areas corresponding to different life stages of fish (e.g. larval, juvenile and adult). This is possible because: i) we can determine the age of the different portions of an otolith; ii) the otolith area ablated by the laser corresponds to a relatively short time lapse (e.g. days to weeks) within the fish life span. The ability to correlate a particular portion of an otolith with a discrete time-point in the life of a fish is one of the most valuable aspects of otolith structure analyses using LA-ICP-MS [5].

A number of papers using LA-ICP-MS have described spatial variability in otolith chemical composition of tropical [10], [19], [20] and temperate fishes [7], [21], [22], (see [5] for a meta-analytical approach), but few studies have investigated patterns of spatial variability at multiple scales. In addition, there is a paucity of information regarding variability in elemental uptake ‘within species’ and heterogeneity in elemental distribution within otoliths (i.e. difference in elemental concentration among areas of an otolith corresponding to the same fish age).

By analyzing the available literature we have found that, in general, little attention has been paid to the use of appropriate experimental designs (*sensu* [23]) in the scientific context of otolith studies. When analyzing otolith chemistry using LA-ICP-MS, the experimental unit is generally the single ablation, with few studies recognizing the importance of within-otolith replication as a key step in assessing variability among otoliths [8], [9], [22]. The presence of within-otolith variability has been acknowledged [1], [12], [24]–[26], but not formally incorporated into experimental designs aimed to assessing differences in elemental fingerprint of fishes from multiple sites on the presumed grounds of: 1) the negligibility of within-otolith variability compared to among-otolith variability [12], [25]; 2) spatial dependence among multiple ablations within an otolith [1], [10], [27].

This approach carries major drawbacks in respect to the quality and potential of the information that is extracted from otoliths. First, knowledge of within-otolith variability is crucial to assessing among-otolith variability, provided that within-otolith measurements can be treated as ‘independent and identically distributed’ replicates. Variability among individuals can then be tested and estimated, based on the within-otolith replication. Second, comparisons of within-otolith variability across spatial scales may provide a fresh insight into the processes that regulate the otolith fingerprint and help to highlight patterns of species dispersal and connectivity.

The purposes of the present study are, therefore, to: i) assess if multiple ablations within an otolith can be used as independent replicates for significance tests (e.g. ANOVA, PERMANOVA) among otoliths, ii) explore the implications of taking into account within-otolith variability while assessing spatial variability in otolith chemistry at multiple spatial scales, iii) investigate homogeneity/heterogeneity in overall variance, iv) assess within-otolith variability across spatial scales, using a multi-factorial experimental design and v) assess the optimal allocation of sampling effort between scales, for testing specific hypotheses in an experimental design.

To accomplish these goals we used a hierarchical sampling design of spatial variation at multiple scales in otolith chemical fingerprints from two Mediterranean coastal fishes (the white sea bream, *Diplodus sargus sargus*, and the two banded sea bream, *Diplodus vulgaris*).

## Materials and Methods

### Ethics statement

The observational study, and the experimental fishing protocol, were carried out at all locations in strict accordance with authorization protocols provided by the Italian Ministry of Agriculture, Foods and Forestry Politics (Permit Number: 0011267-2010). After collection, juvenile individuals were immediately euthanized in an ice slurry (<5°C). The sampling activity did not involve endangered or protected species.

### Study locations and fish collection

**Figure 1:**
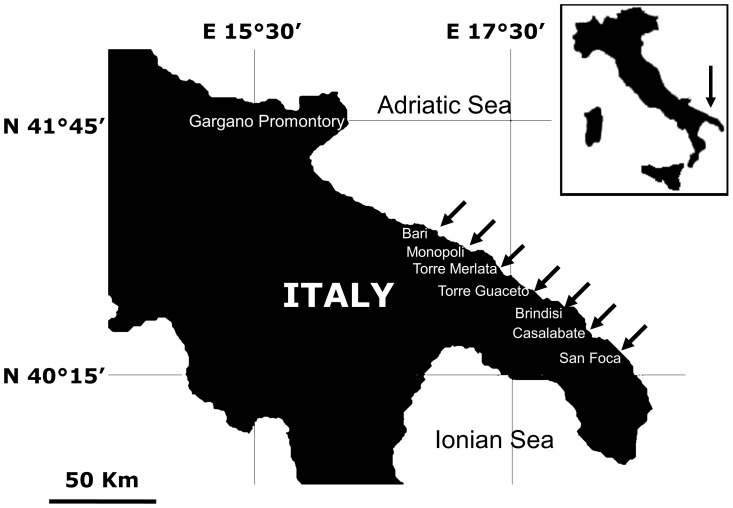
Study area. Sampling locations are indicated with arrows.

We analyzed otoliths from newly settled juveniles of *D. sargus sargus* and of *D. vulgaris*. Juveniles were chosen because of their high density, aggregated distribution and reduced fleeing behavior (i.e. lower escape speed) in comparison with adults [28], these features making them easy to collect. Juveniles of *D. sargus sargus* and *D. vulgaris* were collected along ∼200 km of the Apulian Adriatic coast (Fig. 1), with seven sections of the coast used as sampling locations. Each location was roughly 8 km in length and locations were separated by 15–30 km. Within each location, we randomly selected two sites, each consisting of an embayment with shallow rocky habitats alternating with sandy patches.

Along the overall sampling region, sloping rocky coasts alternate with cliffs and sand/pebble beaches. Generally, the continental shelf is characterized by coastal sands (down to 10–15 m of depth), where extensive *Posidonia oceanica* meadows can be found [29].

Temperature and salinity fields are spatially homogeneous along the studied coast [30], but mesoscale variability in temperature and salinity can be observed in spring and winter, respectively. In addition, small scale variability in both temperature and salinity can be generated by the multitude of run-offs that characterize the area (i.e. from streams and other small freshwater inputs).

We used a hand-net to collect 9–10 individuals per site for *D. sargus sargus* (n = 139) and 10–12 individuals per site for *D. vulgaris* (n = 158), giving a total of 297 individuals. We collected *D. sargus sargus* in late June 2009, and *D. vulgaris* in early May 2010. Sampling was carried out after the settlement peak for each species [28], [31], [32].

*D. sargus sargus* were stored in 95% ethanol and *D. vulgaris* were frozen. Previous work has given inconclusive results about the effect of storage methods (i.e. by freezing or in ethanol) on the chemical composition of otoliths [33], [34]. In this study, for each species, the same storage method was used across locations and sites. Since statistical analyses were carried out separately for each species, we can reasonably exclude any potential bias related to storage method.

### Sample preparation and analysis

In the laboratory, one sagitta was removed from each individual, cleaned of soft tissue using plastic dissecting pins and then mounted sulcus side up onto a glass slide using Crystalbond. A preliminary test was carried out to assess if Crystalbond could be a major source of contamination. We tested if elemental concentrations within Crystalbond were comparable to average otolith concentrations for the two species investigated. The test revealed that, in Crystalbond, Ca was absent and Sr was present with insignificant values in relation to those recorded in otoliths (i.e. lower values in otoliths were 3 orders of magnitude higher than the maximum values recorded in Crystalbond). Mg, Zn, Ba and Pb were present at concentrations comparable to the average for otoliths. Therefore if, despite following standard procedures to avoid any contamination (see later), fragments of Crystalbond remain within otolith crevices, these would have been detectable by abnormally low Ca and Sr estimates.

Otoliths were polished with 3 µm and 1 µm Imperial lapping film to expose inner growth layers for analysis. After polishing with lapping film, otoliths were rinsed and sonicated for 10 minutes in ultra-pure water.

The otoliths were placed in the ablation chamber and viewed remotely on a computer screen where the area for ablation was selected. The laser was focused on the sample surface and fired through the microscope objective lens using a spot size of 30 µm. Each run generally consisted of 40 s acquisition: 10 s blank to correct for background which was subtracted from each sample, 10 s ablation (laser at 65% power, about 6 J/cm^2^) resulting in a pit about 10 µm deep, and 20 s for washout. Prior to analysis, samples were pre-ablated to remove any surface contamination (laser at 50% power). Helium gas was flushed into the ablation cell to reduce the deposition of ablated aerosols and to improve signal intensities. The ablated aerosol was then mixed with Argon before entering the ICP torch. Settings were the same for the two species investigated.

To test whether multiple ablations within each otolith could be considered independent of each other, or whether they displayed an autocorrelation structure within the otolith related to their physical spacing, we ablated 30 pits on two otoliths for each of the two sea bream species (this level of replication is considered to be appropriate for an effective RELATE test of spatial autocorrelation, see later). The pits were placed approximately along the same daily rings (i.e. group of 4–7) at a typical spacing for neighboring pits of about 250–300 µm. The position of each pit in the ablation chamber was recorded in term of its X and Y coordinates.

Having determined that multiple ablations at minimum spacing of approximately 250 µm within an otolith could be treated as independent of each other (see Results section) we used within-otolith pits as replicates for further analyses. All 297 otoliths (the total n for the two species) were analyzed for the chemical composition of the post-settlement segment, namely the region centered approximately on the tenth daily increment beyond the settlement mark (the transition zone in the otolith microstructure corresponding to the settlement of the planktonic larva metamorphosing into a benthic juvenile). In this analysis of spatial scales, we used three horizontal ablation pits per otolith (approximately along the same daily rings) in order to account for within-otolith variability. All otoliths were analyzed using a Thermo Elemental X7 ICP-MS coupled to a NewWave Research UP213 with aperture imaging laser ablation system. External calibration was performed with two Standard Reference Materials (SRM) from the National Institute of Standards and Technology, NIST 610 and NIST 612. We normalized each analyte to ^44^Ca by calculating the ratio of metal to Ca, mass-bias corrected using calibration standards with known analyte to Ca ratios. Calcium was used as an internal standard to account for variation in ablation and aerosol efficiency. Nine elements in *Diplodus sargus sargus* (^7^Li, ^24^Mg, ^55^Mn, ^57^Fe, ^59^Co, ^66^Zn, ^88^Sr, ^138^Ba, ^208^Pb) and 7 in *Diplodus vulgaris* were analyzed (^7^Li, ^24^Mg, ^55^Mn, ^66^Zn, ^88^Sr, ^138^Ba, ^208^Pb). All elements were expressed as ratios relative to ^44^Ca. Detection limits were calculated from the concentration of analyte yielding a signal equivalent to 3× the standard deviation of the blank signal for each of the elements (see Table 1).

**Table 1:** Estimates of precision, accuracy and limits of detection (LOD).

Element	Precision NIST 610 (%)	Precision NIST 612 (%)	Accuracy NIST 610 (%)	Accuracy NIST 612 (%)	LOD
*Diplodus sargus sargus*					
Mg:Ca	8.10	14.5	101	107	0.059
Zn:Ca	7.37	10.34	99	120	0.068
Sr:Ca	4.70	9.43	100	92	0.297
Ba:Ca	8.90	9.84	101	88	0.005
Pb:Ca	13.29	19.07	99	122	0.002
*Diplodus vulgaris*					
Mg:Ca	8.95	15.44	103	110	0.045
Mn:Ca	6.40	10.95	101	113	0.22
Sr:Ca	4.60	10.51	100	93	0.332
Ba:Ca	9.30	9.52	102	89	0.006

LOD are given in mmol mol^−1^. Values for %RSD (% relative standard deviation) and accuracy referred to NISTs are dimensionless. Data are provided separately for the two species because analyzed at different times.

Mean estimates of analytical precision (%RSD, relative standard deviation) and analytical accuracy (observed value/certified reference value in %) for NIST 610 and NIST 612 were calculated based on 100 replicate measurements for *D. sargus sargus* and 109 replicate measurements for *D. vulgaris* (Table 1). In the case of *D. sargus sargus* the recorded values of Li, Mn, Fe and Co were consistently below the detection limits (respectively 0.307, 1.129, 4.071, 0.046 mmol/mol Ca) and, therefore, excluded from the analyses. In the case of *D. vulgaris*, Li, Zn and Pb were consistently below the detection limits (respectively 0.381, 0.040, 0.002 mmol/mol Ca) and, therefore, excluded from the analyses.

### Statistical analyses

#### i) Autocorrelation

The first test was aimed to assessing spatial autocorrelation in the replicates taken along the same daily rings within an otolith. To be treated as replicates for the purposes of testing elemental fingerprint variability among otoliths, using the multivariate permutational methods described below, it is necessary to establish if replicate ablations within an otolith are representative and exchangeable. This is a key step and cannot be assumed without testing. Here, exchangeability implies that there is no tendency for spatial autocorrelation, in which nearby pairs of samples give more similar values than those placed at greater distances. To test the exchangeability we used the RELATE test in the PRIMER software [35], [36], a non-parametric form of Mantel test [37], usually employed to assess trends in time or space, but with good capacity to detect autocorrelation in a multivariate context. In this case, the Spearman rank matrix correlation (ρ) is computed between two resemblance matrices: one constructed as normalized Euclidean distances between the samples of (log-transformed) elemental concentrations for each ablation and the other as (non-normalized) Euclidean distances from the spatial coordinates (X and Y) determining the location of each of the 30 pits sampled. The RELATE null hypothesis of no relationship of elemental composition to spatial position is tested by permuting positional labels among the samples at random, and recalculating the test statistic ρ. If the observed value of ρ is indistinguishable from those generated under random reallocations of the samples to the ablation pit locations, then this is precisely the exchangeability required to validate the use of these samples as replicates in the subsequent permutation tests of differences between otoliths (along with the ‘representativeness’ assumption that the separation of ablation pits effectively covers the spread of available locations along the same daily rings). A RELATE test on 30 pits gives a null hypothesis distribution which could be based on 30! ( = 2.7×10^32^) permutations, a massively large number, allowing even a small degree of spatial autocorrelation to be detectable. In practice, the full permutation distribution is approximated by, for example, 999 or 9999 randomly-drawn permutations but this does not diminish the ability of the test to detect small amounts of spatial autocorrelation, the effectiveness of the test being largely determined by the size of the possible set of permutations, rather than the size of the random selection of these actually calculated (of course, there must be sufficient random selections for the significance level demanded, e.g. a significance level better than p<0.001 could never be achieved from only 999 random permutations).

#### ii) Spatial variability

Given the absence of such autocorrelation at the within-otolith scale (see Results), spatial variability in otolith chemistry was then analyzed using a 3-way unbalanced permutational multivariate analysis of variance (PERMANOVA, [38], [39]). ‘Location’ (Lo) was treated as a random factor (seven levels), ‘Site’ (Si) was treated as a random factor nested in (Lo) (two levels), ‘Otolith’ (Ot) as a random factor nested in (Si) (nine to twelve levels). There were three replicate ablations for each otolith (n = 417 for *D. sargus sargus* and n = 474 for *D. vulgaris*). For each data set, as in the autocorrelation tests described above, the elemental data were first log(x+1) transformed before a normalized Euclidean distance-based similarity matrix was constructed.

Analyses under EXPDES-1 were carried out both for the multi-elemental fingerprint (i.e. multivariate analyses) and for each single element/Ca ratio (i.e. univariate analyses).The utility of this experimental design (hereinafter EXPDES-1) in exploring spatial variability was compared to that of a design lacking within-otolith replication (as largely adopted in the literature, hereinafter EXPDES-2). In the second model, we randomly selected one of the ablations for each otolith and re-ran the analyses, therefore dropping the test for the factor ‘Otolith’ (Ot).

Both multivariate components of variation and the ratio ø =  (estimated magnitude of variance for each factor)/(estimated residual variance) of multi-elemental fingerprint analyses were calculated for the three random factors considered in the PERMANOVAs [17], [22], [40]. Whenever estimated components of variation for a term turned out to be negative, the data were re-analyzed after pooling, i.e. removing that term from the experimental design [41].

To visualize multivariate patterns, non-metric multidimensional scaling (nMDS) ordinations were obtained from Euclidean distance matrices calculated from log(x+1) transformed data, using the data set with three ablations per otolith.

#### iii) Homogeneity of dispersion at site level

We tested the data for homogeneity of overall dispersion at the level of “All Sites” (i.e. the 14 groups from 7 locations by 2 sites) using Permutational Analysis of Multivariate Dispersions (PERMDISP) based on Euclidean distance, which is equivalent to Levene's test for heterogeneity of variances when used on univariate data [39]. PERMDISP is limited to testing a single factor at a time and tests the homogeneity of multivariate dispersions within the levels of that factor based on deviations from the group centroids. To test the factor All Sites, the resemblances entered into PERMDISP were the ‘distances among centroids’ for the three-factor combination Location-Site-Otolith.

#### iv) Within-otolith variability at all scales

To assess within-otolith variability across spatial scales we calculated in PERMDISP the individual deviation values for each pit from centroids of the combined factor Location-Site-Otolith (i.e. the distances, in the normalized Euclidean space, of the individual pits from the centroids of the 3 pits in each otolith). The individual deviation values obtained were then analyzed using PERMANOVA under EXPDES-1 to test for spatial variability in within-otolith dispersion over the hierarchy of scales. This is therefore a simple way of generalizing the Levene's test (as in PERMDISP) to a more complex design structure with several hierarchical levels.

Analyses were carried out for both multi-elemental fingerprints (i.e. multivariate analyses) and for each single element/Ca ratio (i.e. univariate analyses).

#### (v) Optimal allocation of effort among and within otoliths

From the perspective of the assessment of optimal allocation of sampling effort in such a hierarchical experimental design (see [42] for a detailed discussion and formulas), under the assumption that multivariate dispersions are homogeneous at each level of nesting, our results can also be used to establish: i) the optimal number of replicates within an otolith (r*) and ii) the optimal number of otoliths within a site (n*), for a fixed total effort of experimentation. For this, we have used data on 10 otoliths by 3 replicate pits per otolith, separately for the two species. In other words, we have assessed what is the best trade-off among the ‘costs’ of each analysis (in terms of time) and the variance (dispersion) explained. To do this, we estimated the time necessary to prepare an otolith for LA-ICPMS analysis (C1) and that to run a single ablation (C2). C1 includes fish collection, otolith extraction and mounting on glass slide, otolith polishing and sonication (see details in “Sample preparation and analysis” section), giving a total time of approximately 75 minutes (based on this study experience); C2 includes laser positioning and analysis for a total time of approximately 2 minutes. The optimal allocation formula recognizes that, if the 30 samples were equally ‘costly’ to obtain, all 30 would be placed at the higher level (otoliths), but a large disparity in costs between levels (as observed here) could permit a reduction in overall uncertainty at the site level by assigning some effort to reducing the variability propagating through from the within-otolith level.

### Software

All statistical analyses were run using the PRIMER 6 software package [36] with the PERMANOVA+ add-on [39].

## Results

### i) Autocorrelation

**Figure 2:**
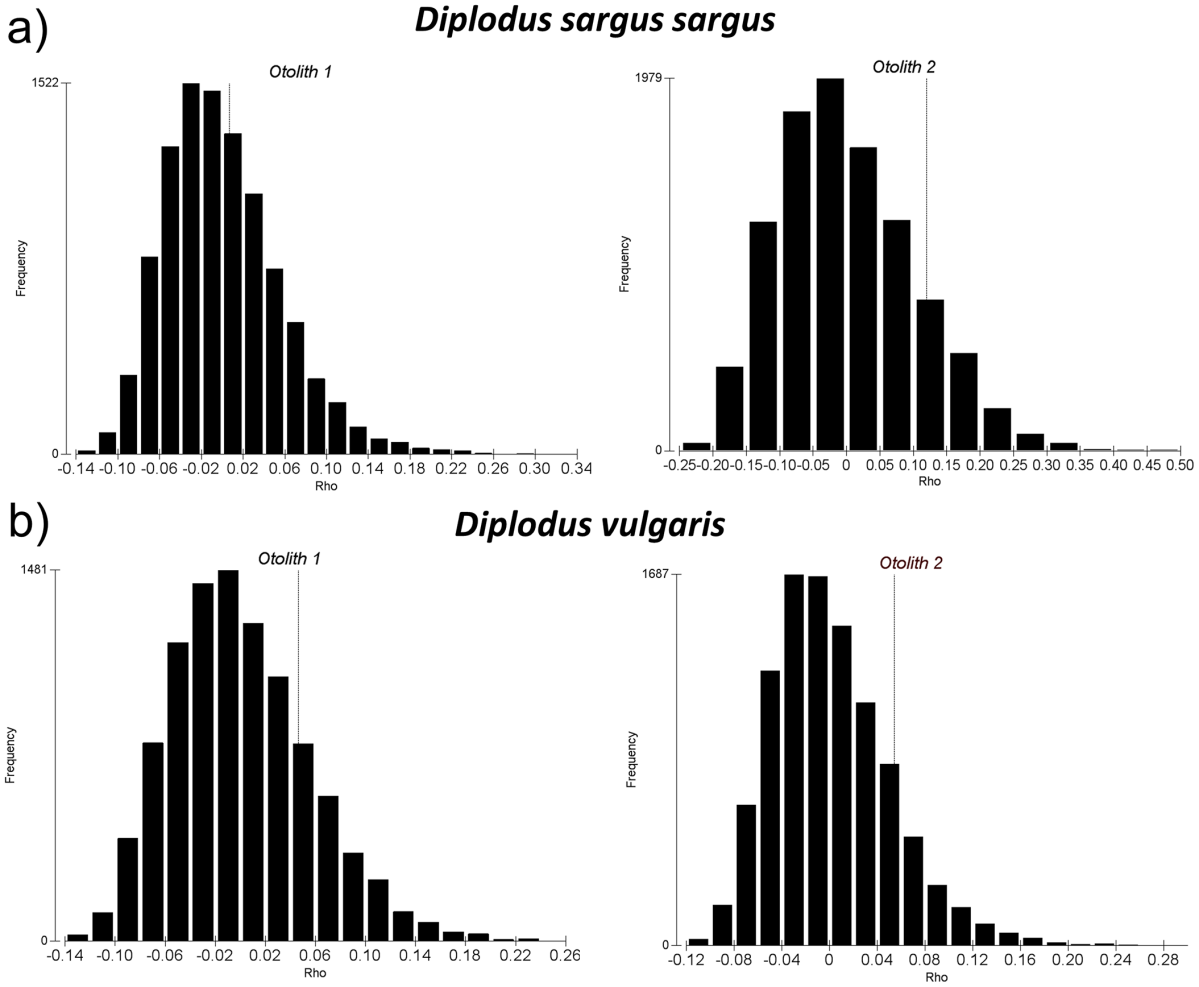
RELATE test for assessment of independence/autocorrelation among multiple ablations on a single otolith for a) *Diplodus sargus sargus* and b) *Diplodus vulgaris*. The frequency distribution of simulated ρ values under the null hypothesis of exchangeability is shown for two otoliths of each of the two species. Vertical line in each graph represents the observed ρ computed for each otolith.

The RELATE test did not provide any evidence of autocorrelation structure among replicates for *Diplodus sargus sargus* (ρ = 0.007; p<40.2% and ρ = 0.12; p<13.2% respectively for the two otoliths examined, Fig. 2a) or *D. vulgaris* (ρ = 0.046; p<13.2% and ρ = 0.054; p<13.6% respectively for the two otoliths examined, Fig. 2b). The non-significant, and near-zero, ρ values validate the use of multiple LA-ICPMS pits on the same otolith as replicates for the subsequent analyses.

### ii) Spatial variability

**Figure 3:**
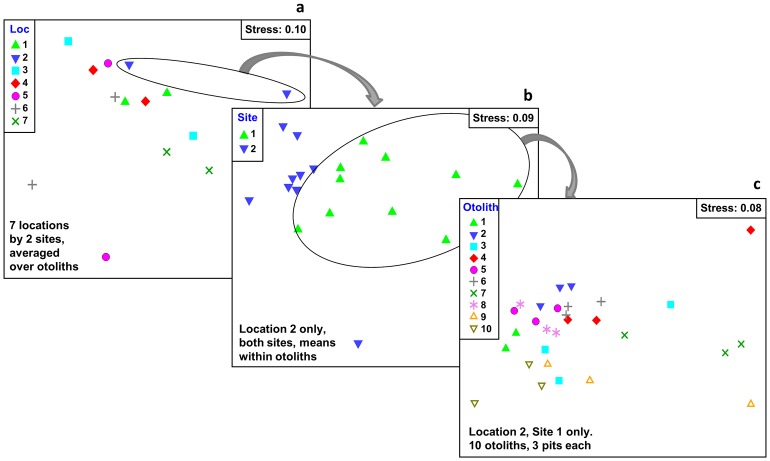
Non-metric MDS plots showing an example of the nested levels of multivariate variation in samples of the multivariate fingerprint for *Diplodus sargus sargus*. The plot is based on log(x+1) transformed data input to normalized Euclidean distance measures. a) all data averaged to the 14 levels of 7 locations with 2 sites per location; b) data for Location 2 only, for the 2 sites and 10 otoliths per site, averaged within otoliths; c) data for Location 2, Site 1 only, for the 10 otoliths and 3 ablation pits per otolith.

Under EXPDES-1, the multivariate chemical composition did not differ among locations but did so at the scale of the site (p<0.001) and at that of the otolith (p<0.001). This pattern was consistent for the two species investigated (Table 2). This pattern is exemplified graphically in a series of three nMDS plots showing the nested levels of variation in the multivariate fingerprint for *Diplodus sargus sargus* (Fig. 3).

**Table 2:** PERMANOVA on data of chemical composition of otoliths under the two experimental designs EXPDES-1 (incorporating three ablations per otolith and so having Otolith as a factor) and EXPDES-2 (using only one ablation per otolith and thus without Otolith as a factor).

*EXPDES-1*						
	*Diplodus sargus sargus*			*Diplodus vulgaris*		
Source	d.f.	MS	pF	d.f.	MS	pF
Lo	6	17.99	0.82 ns	6	0.0372	0.34 ns
Si(Lo)	7	21.86	4.85***	7	0.1078	7.82***
Ot(Si(Lo))	125	4.50	6.18***	143	0.0138	3.81***
Res	277	0.72		317	0.0036	
Total	415			473		

*EXPDES-2*						
	*Diplodus sargus sargus*			*Diplodus vulgaris*		
Source	d.f.	MS	pF	d.f.	MS	pF
Lo	6	7.96	0.81 ns	6	0.0145	0.39 ns
Si(Lo)	7	9.76	4.78***	7	0.0363	4.84***
Res	125	2.04		144	0.0075	
Total	138			157		

pF =  Pseudo-F. ns: not significant;

***: significant at p<0.001. Lo =  locations, Si =  sites (nested in locations), Ot =  otoliths (nested in sites).

**Figure 4:**
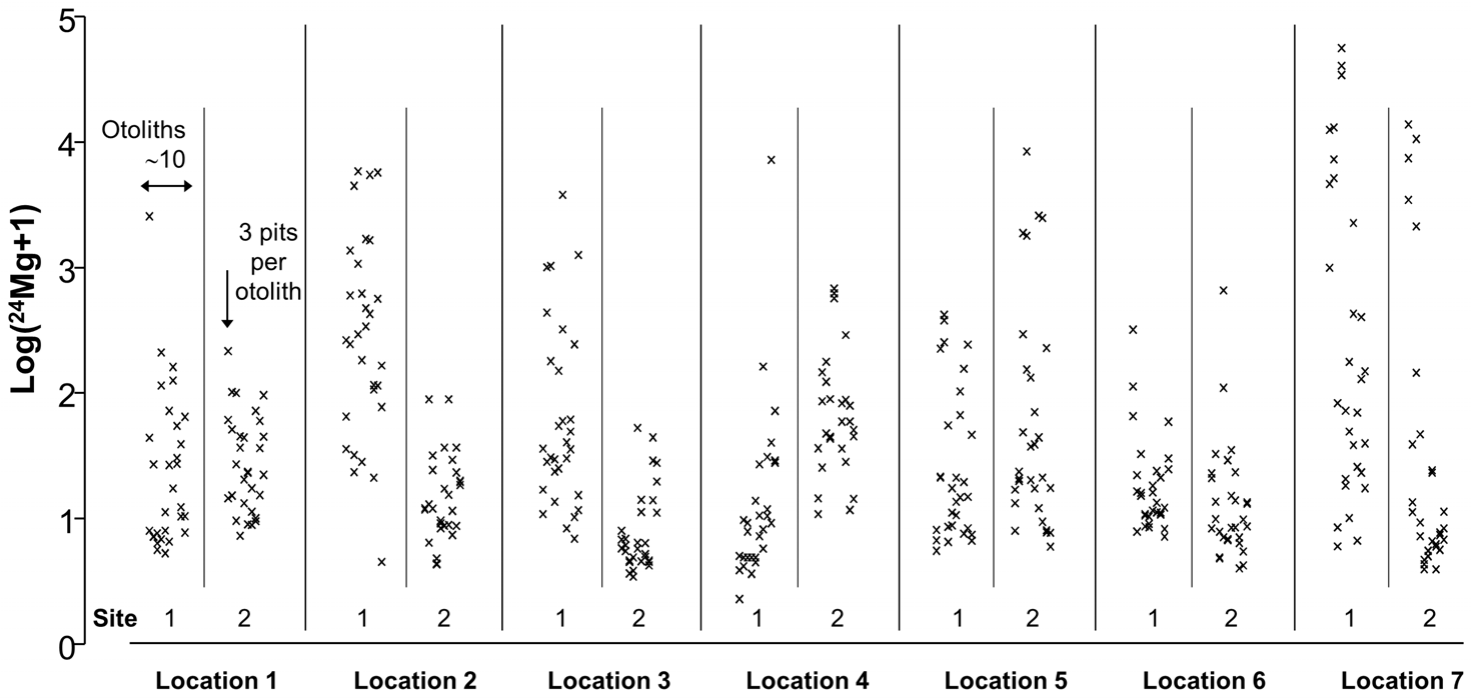
Example of log transformed data for a single variable (24 Mg/Ca) measured in *Diplodus sargus sargus* from individual ablation pits: 3 pits per otolith, 8–12 otoliths per site, 2 sites per location and 7 locations.

In *Diplodus sargus sargus*, univariate analyses for Sr/Ca, Zn/Ca, Mg/Ca and Pb/Ca generated the same pattern shown by the multivariate fingerprint, with significant variability detected at the scale of site and otolith (Table S1), while Ba/Ca showed significant variability only among otoliths (Table S1). All the elemental ratios did not differ among locations. A graphical example of this pattern is provided for Mg/Ca in *Diplodus sargus sargus* (Fig. 4). Univariate analyses in *Diplodus vulgaris* showed that Mn/Ca and Mg/Ca differed significantly at both the scale of site and otolith (Table S2) while Sr/Ca and Ba/Ca showed significant variability only at the scale of the otolith (Table S2). All the elemental ratios did not differ among locations.

For multi-elemental composition of both species, a sampling design incorporating one ablation per otolith (EXPDES-2) allowed the detection of significant differences at the scale of the site (p<0.001), but not at the scale of the location (Table 2). For EXPDES-2, the pseudo-*F* test used to test for significant variability at the scale of sites was constructed by dividing the Mean Square (MS) of the factor Site by the Residual MS, the latter estimating variance among single pits from each otolith used in the experiment.

Although it could be argued that the test for the Site effect (with 9–12 otoliths per site and 14 sites) will have greater power than that for the Location (with 2 sites at 7 locations), the estimated components of variation turned out to be negative for factor Lo, both in EXPDES-1 and EXPDES-2. This strongly indicates that the lack of significance of Location was not due to a lack of power but to a genuine absence of the effect; data were, therefore, re-analyzed after pooling for the factor Lo, by means of a 2-way nested design (14 sites, 9–12 otoliths per site and 3 pits per otolith). For EXPDES-1, most of the variability was associated with the pooled factor Site, both for *D. sargus sargus* (ø = 2.68; 53.7% of total variation) and *D. vulgaris* (ø = 2.38; 54.9% of total variation). In *D. vulgaris*, the remaining variability was almost equally partitioned between the Otolith (ø = 0.96; 22.2% of total variation) and the Residual (i.e., within single otoliths, 23.0% of total variation). In *D. sargus sargus*, 26.3% of the total variation was associated with the Otolith (ø = 1.31) and 20.0% with the Residual.

For EXPDES-2, after pooling, most of the variability was associated with V(Res), here representing variation between plus variation within otoliths at each site, both for *D. sargus sargus* (63.0%) and *D. vulgaris* (67.9%). The pooled Site factor was, therefore, responsible for 37.0% of total variation (ø = 0.58) in *D. sargus sargus* and 32.1% (ø = 0.47) in *D. vulgaris*.

### iii) Homogeneity of dispersion at site level

PERMDISP showed that patterns of dispersion for the factor All Sites differed between fish species: there was no evidence of departure from homogeneity for *D. vulgaris*, while there was significant heterogeneity in *D. sargus sargus* (p<0.001).

### iv) Within-otolith variability at all scales

Within-otolith variability differed between the two species: PERMANOVA results showed that within-otolith dispersion in *D. vulgaris* did not vary at the scale of the site and location but did among otoliths. In contrast, in *D. sargus sargus*, within-otolith dispersion differed significantly at the scale of the site and among otoliths, but again not at that of location (Table 3). In fact, none of the statistical tests detected any effects at the scale of the location: the sampling design reverts effectively to the simpler 2-way nested design of within-otolith readings from multiple otoliths collected at 14 sites.

**Table 3:** PERMANOVA on data of within-otolith dispersion of otolith chemical composition (obtained from PERMDISP) under the design EXPDES-1 (incorporating three ablations per otolith and so having Otolith as a factor).

	*Diplodus sargus sargus*			*Diplodus vulgaris*		
Source	d.f.	MS	pF	d.f.	MS	pF
Lo	6	0.751	0.35 ns	6	0.000483	0.17 ns
Si(Lo)	7	2.158	4.78***	7	0.002880	7.82 ns
Ot(Si(Lo))	125	0.451	3.49***	143	0.001490	3.81***
Res	277	0.129		317	0.000539	
Total	415			473		

pF =  Pseudo-F. ns: not significant;

***: significant at p<0.001. Lo =  locations, Si =  sites (nested in locations), Ot =  otoliths (nested in sites).

In *Diplodus sargus sargus*, univariate analyses for Zn/Ca, Mg/Ca revealed the same pattern as for the multivariate fingerprint, with significant variability detected at the scale of the site and the otolith (Table S3), while Ba/Ca, Sr/Ca and Pb/Ca showed significant variability only among otoliths and not among sites (Table S3). All the elemental ratios did not differ among locations.

Univariate analyses in *Diplodus vulgaris* showed that all the elemental ratios (Mg/Ca, Mn/Ca, Sr/Ca, Ba/Ca) differed significantly among otoliths, but not at the site or location level (Table S4).

### (v) Optimal allocation of effort among and within otoliths

Based on the components of variation estimated from multivariate PERMANOVA under EXPDES-1, the optimal allocation of sampling effort for a fixed time expenditure corresponds to r* (i.e. optimal number of replicates within an otolith)  = 2.8 and n* (i.e. optimal number of otoliths within a site) = 10.1 for *D. sargus sargus* and r* = 2.2 and n* = 10.2 for *D. vulgaris*. This indicates that our choice of r = 3 and n around 9 to 12 was an effective balance of effort for this experimental design.

## Discussion

The main findings of this work are: i) multiple ablations along the same daily rings within an otolith do not necessarily imply spatial dependency (so that this procedure can be used to estimate residual variability in a hierarchical sampling design); ii) taking into account within-otolith variability allowed a demonstration that individuals at the same site may show a significant variability in elemental uptake; iii) patterns of multivariate dispersion in the otolith fingerprint, at higher than the within-otolith level, are inconsistent between species; iv) analysis of within-otolith variability across spatial scales also showed differences between the two species; v) as a result of the high time-cost of preparing each otolith, and the relatively low cost of running multiple ablations, optimal allocation of sampling effort should typically include some level of within-otolith replication in the experimental design.

The possibility of ‘non-independence’ among multiple ablations within each otolith cannot be asserted *a priori*, i.e. without formal testing. Some authors (e.g. [1], [10], [27]) state that the dependence of chemical measures within each otolith is implicit in the fact that the otolith grows within the same fish, and is exposed to the same physiological conditions and the same ‘internal’ environment. However, to draw a logical parallel, abundance estimates of fish densities obtained through replicated visual censuses at a site (i.e. a stretch of coast) are also influenced by a number of ‘shared’ environmental variables (e.g. water temperature, clarity and salinity, or wave/wind exposure, trophic resources, habitat complexity or heterogeneity typical of that site), but only the use of formal procedures testing for autocorrelation are likely to determine the minimum distance at which replicated transects must be located in order to avoid spatially dependent measures [43]. Similar reasoning should be applied to multiple ablations from a single fish. In this paper, we provide evidence that multiple ablations within an otolith can, in this case, be treated as independent replicates in a nested sampling design. This study provides, however, a preliminary view and further studies are required to assess unambiguously the minimum distance needed to consider within-otolith replicates as exchangeable in standard permutation testing.

To the best of our knowledge, our study is the first attempting to formally test the variability in otolith fingerprints among individuals collected at the same site. We observed significant variability among individuals in both species, indicating that elemental uptake processes can vary among organisms of the same species exposed to the same environmental conditions (i.e. collected at the same site). This evidence can be related to among-individual variability in physiological processes and to possible differences in feeding, both factors potentially affecting elemental uptake in fish [1], [2], [10]. It has to be highlighted that significant variability among individuals was recorded both for the overall multi-elemental fingerprint and for single elemental ratio in both species. From this perspective, we did not see consistent evidence of different patterns between elements that are generally considered as more influenced by the environment (e.g., Sr and Ba) and elements under physiological regulation (e.g., Zn and Mn), though no significant spatial variability (at the location or site scale) was evident for Ba in either species.

In both species, about one-fifth of the total variance is attributable to within-otolith variability. This identifies a non-negligible source of variability in elemental composition among “contemporary” (i.e. placed approximately along the same daily rings) areas of each individual otolith. To the best of our knowledge, only [24] formally tested for within-otolith variability, finding significant differences between otolith areas corresponding to the same fish age, but using a full repeated measures design on single elements. Our study supports graphical evidence from 2D maps of elemental concentrations, created through x-ray fluorescence spectrometry, indicating heterogeneity in elemental distributions within otoliths [44], [45], [46]. Thus, a single laser ablation (spot/transect) within an otolith may not truly represent the history of elemental exposure.

Different patterns of multivariate dispersion among all sites were found in the two species investigated, suggesting that different ecological/environmental processes (e.g. physiological, dispersive) can occur in the two species. In *D. sargus sargus*, we found significant heterogeneity among sites, with potential, and not mutually exclusive, explanations involving: i) variability in site fidelity at a very early stage (first days after settlement and before collection), with individuals collected in a single site coming from multiple settlement sites and determining homogeneity or heterogeneity in variance according to the magnitude of environmental variability experienced over a larger spatial scale (the one encompassing both sampling site and settlement sites); ii) high short-term temporal variability in environmental conditions after settlement, with otolith areas corresponding in all individuals approximately to 10 days after settlement, potentially corresponding to different calendar days depending on settlement date of each individual; iii) intrinsic variability in the uptake process among individuals. Nevertheless, the first two processes appear unlikely because a) *D. sargus sargus* were collected shortly after settlement [31], [32], excluding extensive displacements of very early juveniles in a week (the maximum temporal range included in a single ablation), and b) individuals collected presented low within-site variability in settlement date (estimated through otolith analyses, Di Franco and Guidetti unpublished data), reducing the possibility of differential effects of temporal variability within each site. From this perspective, the intrinsic variability in the uptake process among individuals seems to be the most likely mechanism, strengthening our finding of significant inter-individual variability. On the other hand in *D. vulgaris*, we did not find any significant heterogeneity among sites.

Within-otolith variation in elemental composition can be related to the otolith crystalline structure, determined during biomineralization (the process of otolith formation and accretion, [47]). Otolith bio-mineralization is a temperature-dependent process [48] and higher temperatures determine faster growth and increased numbers of crystal defects. This process can induce greater entrapment of trace element impurities into the growing crystal [10] and likely higher within-otolith variability. From this perspective we can hypothesize that: 1) variability in temperature (e.g. among sites) could induce differences in within otolith element variability, and 2) fishes growing at higher temperature will have higher within otolith elemental variability. *D. sargus sargus* settles in late spring in the study area [31], while *D. vulgaris* settles in winter [32]. During spring, the south Adriatic sea shows a marked spatial variability in sea surface temperature [30] that, according to our hypothesis, could determine the significant variability detected in *D. sargus sargus*. On the other hand, more stable thermal conditions occur in winter and this pattern potentially may result in greater homogeneity in *D. vulgaris*. The hypothesis of an effect of temperature on within-otolith elemental variability seems to be strengthened by the finding of higher average within-otolith dispersion in *D. sargus sargus* compared to *D. vulgaris* (data not reported), with the juveniles of *D. sargus sargus* growing at higher temperature. This difference arises both comparing within-otolith dispersion calculated from different pools of elements for the two species (as presented in Materials and Methods) and from a pool of the three common elements (Sr, Ba and Mg). However, our hypothesis about the relationship between temperature and within-otolith dispersion should be considered tentative because other environmental/physiological variables (e.g. maternal effect) could affect this process.

Additional sources of disturbance inducing physiological stress in fish could involve, amongst other possibilities, the density of individuals at each site/location and the density of their predators. For *Diplodus sargus sargus* significant variability in density has been recorded at a small spatial scale [28], whilst further data show no evidence of such variability in *Diplodus vulgaris* (authors' unpublished data). No information is available, however, about type and density of predators for the two species at a juvenile stage in the study area.

Due to the poor understanding of biomineralization mechanisms [49], [50] and the novelty of our findings, it is challenging to provide stringent explanations of the possible mechanisms determining these patterns. If ascertained as a marker of environmental conditions experienced by a fish at a given time, within-otolith variability could be useful in revealing fish exposure to stressful conditions (e.g. acidification, extreme temperatures or pollutants) as detected for other otolith variables as size, shape and symmetry [50], [51], [52]. Environmental stress can induce developmental instability (the inability of an organism to compensate for disturbances during development, [53]) and increased ‘noise’, producing, as a consequence, lower developmental precision in morphological traits [54].

In this study, we did not find any difference in patterns of variability at multiple spatial scales between the experimental designs using single and multiple ablations within an otolith. Thus, the addition of the factor “otolith” in the experimental design does not make any radical improvement to our ability to detect patterns across spatial scales (i.e. sites and locations). Indeed, we would not expect it to, since a key determinant of the power to test patterns at a higher level in a nested design is represented by the number of replicates taken at the level immediately below. Nonetheless, there is an advantage for optimal allocation of sampling effort, in a case such as this, in taking within-otolith observations, where ‘costs’ of such repeats are much less than for repeat otoliths, because a (minor) part of the observed variance at a higher level can be reduced at little cost. Further advantages of sampling at this lowest scale of variability include the testing and scaling of variability among individuals and the examination of intra-otolith variability at different spatial scales, potentially providing an insight into stressful environmental conditions experienced by each individual.

Researchers recognize otolith microchemistry as a helpful tool in obtaining data on a number of ecological processes (e.g. natal origin, larval dispersal, juvenile and adult connectivity, etc.) and in promoting sound fisheries management. Whilst our ability to obtain information from otolith elemental composition continues to increase through the implementation of new analytical techniques (e.g. x-ray fluorescence spectrometry), we have to acknowledge some potential limitations (e.g. lack of information about among-individual variability in elemental uptake, within-otolith variability). In this study, we provide some novel evidence that may contribute to refining sampling designs and to advancing our general understanding of the mechanisms regulating elemental fingerprints, with the ultimate purpose of further developing experimental frameworks for assessing patterns of dispersal/connectivity of coastal fishes.

## Supporting Information

Table S1 **Univariate PERMANOVA analyses on single chemical elements of** ***Diplodus sargus sargus*** **.**
(DOCX)

Table S2 **Univariate PERMANOVA analyses on single chemical element of** ***Diplodus vulgaris*** **.**
(DOCX)

Table S3 **PERMANOVA on data of within-otolith dispersion of single chemical element otolith composition (obtained from PERMDISP) of** ***Diplodus sargus sargus*** **.**
(DOCX)

Table S4 **PERMANOVA on data of within-otolith dispersion of single chemical element otolith composition (obtained from PERMDISP) of** ***Diplodus vulgaris*** **.**
(DOCX)
