# Supplementary material for: Within-Otolith Variability in Chemical Fingerprints: Implications for Sampling Designs and Possible Environmental Interpretation
Source: PLoS One. 2014 Jul 7;9(7):e101701. doi: 10.1371/journal.pone.0101701 (PMC4085012; doi:10.1371/journal.pone.0101701)
Supplement: Table S1 — Univariate PERMANOVA analyses on single chemical elements of Diplodus sargus sargus . (DOCX) [file pone.0101701.s001.docx]

**Table S1. Univariate PERMANOVA analyses on single chemical elemental ratios of *Diplodus sargus sargus* under the experimental designs EXPDES-1 (incorporating three ablations per otolith and so having Otolith as a factor).** pF = Pseudo-F. ns: not significant; ***: significant at p < 0.001. Lo = locations, Si = sites (nested in locations), Ot = otoliths (nested in sites).

|  |  | Mg/Ca | | Zn/Ca | | Ba/Ca | | Sr/Ca | | Pb/Ca | |
| --- | --- | --- | --- | --- | --- | --- | --- | --- | --- | --- | --- |
| Source | d.f. | MS | pF | MS | pF | MS | pF | MS | pF | MS | pF |
| Lo | 6 | 15.48 | 0.79ns | 0.24 | 0.63ns | 2.41E-2 | 0.92ns | 2.23 | 1.088ns | 5.19E-3 | 1.27ns |
| Si(Lo) | 7 | 19.40 | 5.08*** | 0.38 | 4.11ns | 2.61E-2 | 1.18ns | 2.05 | 3.60*** | 4.06E-3 | 3.02*** |
| Ot(Si(Lo)) | 125 | 3.82 | 5.53*** | 9.34E-2 | 5.31ns | 2.21E-2 | 43.89*** | 0.56 | 28.59*** | 1.34E-3 | 6.90*** |
| Res | 277 | 0.69 |  | 1.75E-2 |  | 5.04E-4 |  | 1.99E-2 |  | 1.95E-4 |  |
| Total | 415 |  |  |  |  |  |  |  |  |  |  |
