# Supplementary material for: Within-Otolith Variability in Chemical Fingerprints: Implications for Sampling Designs and Possible Environmental Interpretation
Source: PLoS One. 2014 Jul 7;9(7):e101701. doi: 10.1371/journal.pone.0101701 (PMC4085012; doi:10.1371/journal.pone.0101701)
Supplement: Table S2 — Univariate PERMANOVA analyses on single chemical element of Diplodus vulgaris . (DOCX) [file pone.0101701.s002.docx]

**Table S2. Univariate PERMANOVA analyses on single chemical elemental ratios of *Diplodus vulgaris* under the experimental designs EXPDES-1 (incorporating three ablations per otolith and so having Otolith as a factor).** pF = Pseudo-F. ns: not significant; *: significant at p < 0.05; ***: significant at p < 0.001. Lo = locations, Si = sites (nested in locations), Ot = otoliths (nested in sites).

|  |  | Mg/Ca | | Mn/Ca | | Ba/Ca | | Sr/Ca | |
| --- | --- | --- | --- | --- | --- | --- | --- | --- | --- |
| Source | d.f. | MS | pF | MS | pF | MS | pF | MS | pF |
| Lo | 6 | 2.80E-2 | 0.28ns | 1.77E-4 | 1.61ns | 1.96E-4 | 2.49ns | 8.51E-3 | 0.80ns |
| Si(Lo) | 7 | 9.69E-2 | 12.41*** | 1.10E-4 | 2.52* | 7.85E-5 | 1.37ns | 1.066E-2 | 1.79ns |
| Ot(Si(Lo)) | 143 | 7.80E-3 | 2.55*** | 4.39E-5 | 5.50*** | 5.74E-5 | 1.21* | 5.94E-3 | 11.90*** |
| Res | 317 | 3.05E-3 |  | 7.98E-6 |  | 4.76E-5 |  | 4.99E-4 |  |
| Total | 473 |  |  |  |  |  |  |  |  |
