# Supplementary material for: Within-Otolith Variability in Chemical Fingerprints: Implications for Sampling Designs and Possible Environmental Interpretation
Source: PLoS One. 2014 Jul 7;9(7):e101701. doi: 10.1371/journal.pone.0101701 (PMC4085012; doi:10.1371/journal.pone.0101701)
Supplement: Table S3 — PERMANOVA on data of within-otolith dispersion of single chemical element otolith composition (obtained from PERMDISP) of Diplodus sargus sargus . (DOCX) [file pone.0101701.s003.docx]

**Table S3. PERMANOVA on data of within-otolith dispersion of single elemental ratios (obtained from PERMDISP) of *Diplodus sargus sargus* under the design EXPDES-1 (incorporating three ablations per otolith and so having Otolith as a factor).** pF = Pseudo-F. ns: not significant; *: significant at p < 0.05; ***: significant at p < 0.001. Lo = locations, Si = sites (nested in locations), Ot = otoliths (nested in sites).

|  |  | Mg/Ca | | Zn/Ca | | Ba/Ca | | Sr/Ca | | Pb/Ca | |
| --- | --- | --- | --- | --- | --- | --- | --- | --- | --- | --- | --- |
| Source | d.f. | MS | pF | MS | pF | MS | pF | MS | pF | MS | pF |
| Lo | 6 | 0.96 | 0.42ns | 6.03E-3 | 0.18ns | 4.54E-4 | 0.58ns | 9.85E-3 | 0.80ns | 1.97E-4 | 1.19ns |
| Si(Lo) | 7 | 2.24 | 4.85*** | 3.32E-2 | 2.31* | 7.76E-4 | 1.45ns | 1.22E-2 | 0.75ns | 1.65E-4 | 1.11ns |
| Ot(Si(Lo)) | 125 | 0.46 | 3.43*** | 1.43E-2 | 5.23*** | 5.34E-4 | 5.01*** | 1.62E-2 | 4.93*** | 1.49E-4 | 4.19*** |
| Res | 277 | 0.13 |  | 2.74E-3 |  | 1.06E-4 |  | 3.28E-3 |  | 3.56E-5 |  |
| Total | 415 |  |  |  |  |  |  |  |  |  |  |
