# Supplementary material for: Within-Otolith Variability in Chemical Fingerprints: Implications for Sampling Designs and Possible Environmental Interpretation
Source: PLoS One. 2014 Jul 7;9(7):e101701. doi: 10.1371/journal.pone.0101701 (PMC4085012; doi:10.1371/journal.pone.0101701)
Supplement: Table S4 — PERMANOVA on data of within-otolith dispersion of single chemical element otolith composition (obtained from PERMDISP) of Diplodus vulgaris . (DOCX) [file pone.0101701.s004.docx]

**Table S4. PERMANOVA on data of within-otolith dispersion of single elemental ratios (obtained from PERMDISP) of *Diplodus vulgaris* under the design EXPDES-1 (incorporating three ablations per otolith and so having Otolith as a factor).** pF = Pseudo-F. ns: not significant; ***: significant at p < 0.001. Lo = locations, Si = sites (nested in locations), Ot = otoliths (nested in sites)

|  |  | Mg/Ca | | Mn/Ca | | Ba/Ca | | Sr/Ca | |
| --- | --- | --- | --- | --- | --- | --- | --- | --- | --- |
| Source | d.f. | MS | pF | MS | pF | MS | pF | MS | pF |
| Lo | 6 | 1.19E-3 | 0.39ns | 9.22E-6 | 1.68ns | 6.65E-5 | 0.83ns | 2.58E-4 | 0.96ns |
| Si(Lo) | 7 | 3.05E-3 | 1.92ns | 5.49E-6 | 0.76ns | 8.01E-5 | 1.08ns | 2.68E-4 | 1.04ns |
| Ot(Si(Lo)) | 143 | 1.59E-3 | 2.89*** | 7.22E-6 | 6.22*** | 7.44E-5 | 13.28*** | 2.58E-4 | 2.72*** |
| Res | 317 | 5.51E-4 |  | 1.16E-6 |  | 5.60E-6 |  | 9.47E-5 |  |
| Total | 473 |  |  |  |  |  |  |  |  |
